# Cystic biliary atresia with paucity of bile ducts and gene mutation in KDM6A: a case report

**DOI:** 10.1186/s40792-019-0688-4

**Published:** 2019-08-14

**Authors:** Daisuke Masui, Suguru Fukahori, Tatsuki Mizuochi, Yoriko Watanabe, Kaori Fukui, Shinji Ishii, Nobuyuki Saikusa, Naoki Hashizume, Naruki Higashidate, Saki Sakamoto, Aiko Takato, Koh-ichiro Yoshiura, Yoshiaki Tanaka, Minoru Yagi

**Affiliations:** 1 Department of Pediatric Surgery, Kurume University School of Medicine, 67 Asahi-machi, Kurume, 830-0011 Japan; 2 Department of Pediatrics and Child Health, Kurume University School of Medicine, Kurume, Japan; 3 Research Institute of Medical Mass Spectrometry, Kurume University School of Medicine, Kurume, Japan; 4 Department of Human Genetics, Nagasaki University Graduate School of Biomedical Sciences, Nagasaki, Japan; 5 Division of Medical Safety Management, Kurume University School of Medicine, Kurume, Japan

**Keywords:** Biliary atresia, *KDM6A*, Paucity of bile ducts, Kabuki syndrome, Neonate

## Abstract

**Background:**

Biliary atresia (BA) cases are generally not associated with congenital abnormalities. However, accurate diagnosis of BA is often challenging because the histopathological features of BA overlap with those of other pediatric liver diseases and rarely overlap with those of other genetic disorders. We experienced a rare case of BA with the histopathological finding of bile duct paucity, a gene mutation in *KDM6A*, and KS-like phenotypes.

**Case presentation:**

A male baby was diagnosed with biliary atresia by intraoperative cholangiography at 4 days of age, and histological examination following a liver biopsy revealed a paucity of bile ducts and several typical clinical findings of Alagille syndrome. However, Alagille syndrome was ruled out after neither *JAG1* nor *NOTCH2* gene mutations were identified. Whole-exome sequencing on DNA from his parents was additionally performed to examine other possible syndromic disorders, and a mutation was identified in *KDM6A*. However, Kabuki syndrome was not diagnosed as a result.

The histological finding of interlobular bile duct paucity and the genetic mutation in *KDM6A*, as well as several clinical findings consistent with Alagille syndrome or Kabuki syndrome, made it difficult to confirm the diagnosis of BA.

**Conclusions:**

Based on the interesting findings of the present case, we hypothesized that *KDM6A* is associated with hepatic malformations via a connection with the Notch signaling pathway.

## Background

Biliary atresia (BA) cases are generally not associated with the monogenic disorder. However, accurate diagnosis of BA is often challenging because the histopathological features of BA overlap with those of other pediatric liver diseases and rarely overlap with those of other genetic disorders and syndromes [1–3].

Kabuki syndrome (KS) is a rare, multiple congenital anomaly syndrome demonstrating several distinctive clinical findings, and with identified gene mutations in *KMT2D* on chromosome 12 and *KDM6A* on chromosome X [4].

The prevalence of KS has been estimated to be 1 in 32,000 Japanese newborn infants [5], and congenital hepatic abnormalities such as BA, neonatal sclerosing cholangitis, and idiopathic hepatic fibrosis have been sporadically described in patients with KS [6–9].

There have been a few reports on KS accompanying BA despite no gene examinations [7, 9, 10]. Herein, we report a rare case of BA with the histopathological finding of bile duct paucity, a gene mutation in *KDM6A*, and KS-like phenotypes.

## Case presentation

**Fig. 1:**
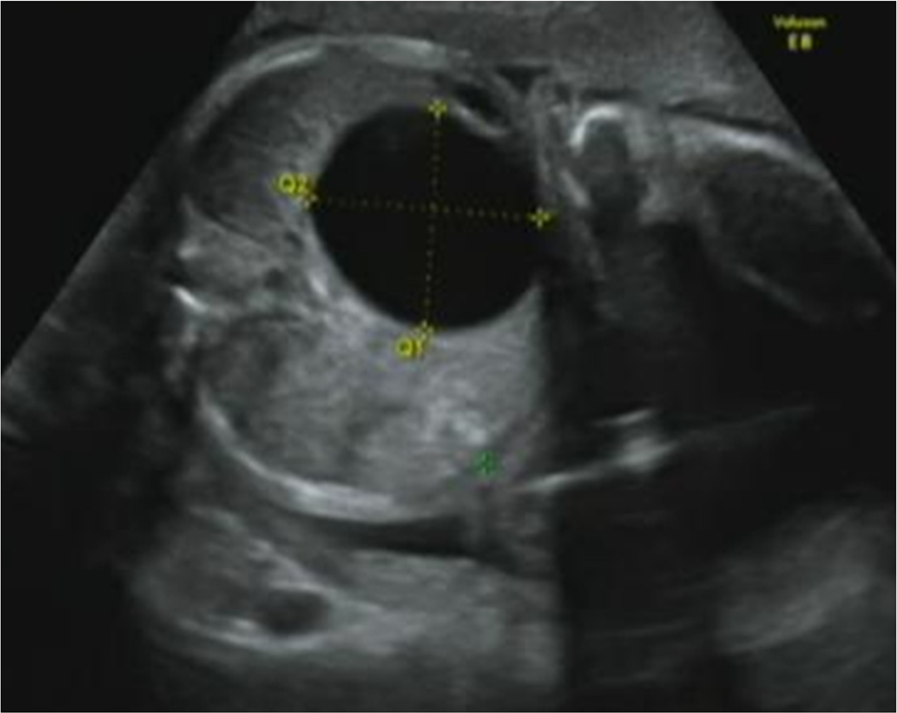
Antenatal ultrasonographic findings at 16 weeks gestation. A cystic lesion, 37 × 40 mm in size, was observed in the hepatic hilum

**Fig. 2:**
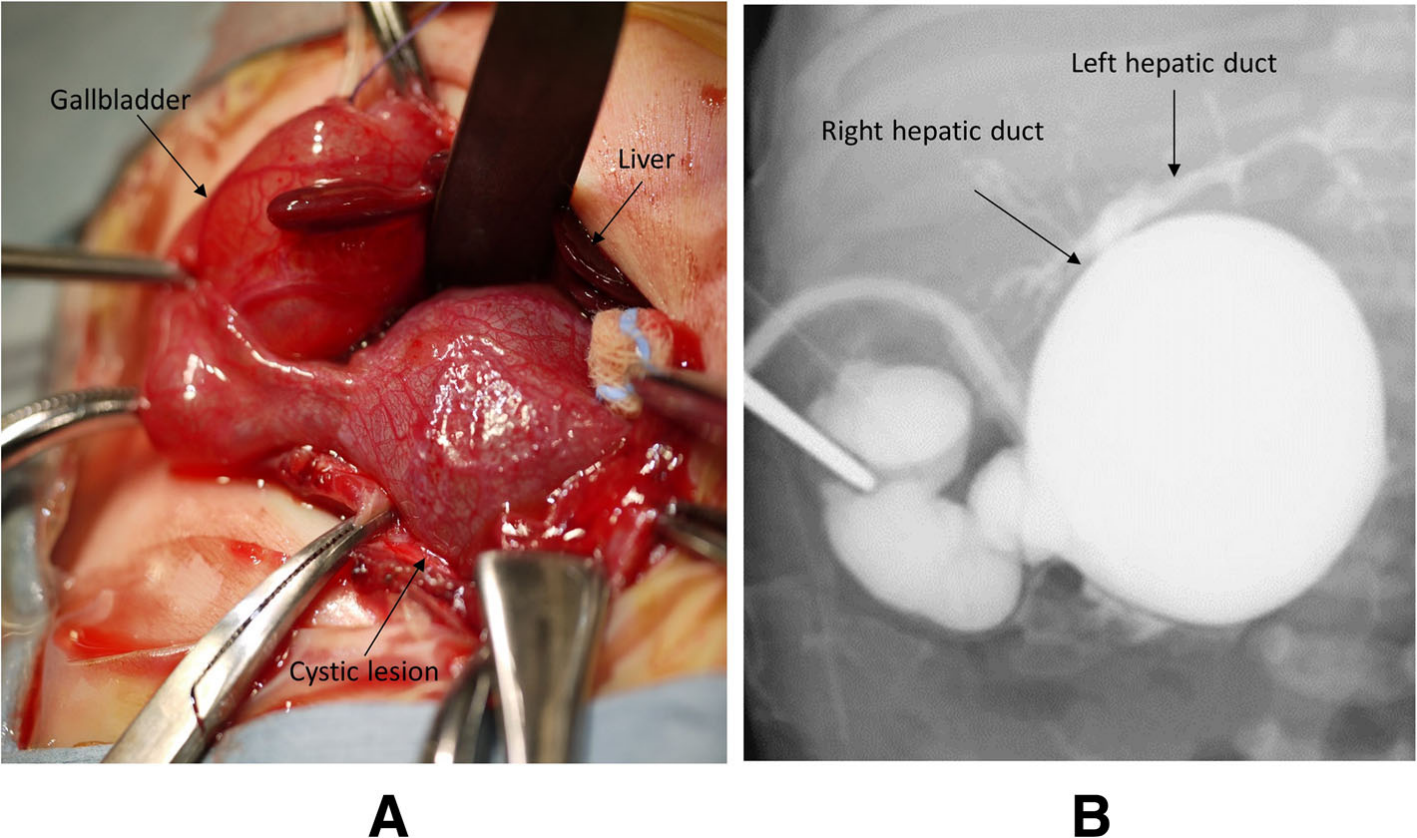
**a**, **b** Intraoperative cholangiography. A cystic mass at the porta hepatis with connection to a tree pattern of intrahepatic ductules, and no connection to the intestinal lumen was found during the intraoperative cholangiography

A male baby weighing 3330 g was delivered at 39 weeks gestation by normal vaginal delivery. The family history was unremarkable. The prenatal ultrasound at 16 weeks gestation demonstrated a cystic lesion, 37 × 40 mm in size, in the fetus’ abdomen (Fig. 1) beneath the liver, suggesting the diagnosis of a choledochal cyst or cystic BA. The fetal karyotype was normal (46, XY). Jaundice and acholic stools were observed when the neonate was 1 day old. Laboratory findings revealed elevated total bilirubin (6.24 mg/dl), direct bilirubin (4.53 mg/dl), AST (124 IU/l), ALT (65 IU/l), γ-GTP (47 IU/l), and TBA (139.2 μmol/l); appropriate tests looking for viral infection such as cytomegalovirus and herpes simplex virus were negative. Ultrasonography revealed a cystic mass, 43 × 32 mm in size, at the porta hepatis, without dilatation of the intrahepatic bile duct. The postnatal examinations also suggested the diagnosis of cystic BA or choledochal cyst. Thus, an exploratory laparotomy was performed at 4 days of age. Intraoperative cholangiography showed a tree pattern of intrahepatic bile ductules (Nio’s classification), a cystic common bile duct, and no connection to the intestinal lumen (Fig. 2a, b). There was no connection from common bile duct to the intestinal lumen by intraoperative repeating cholangiography, and the complete closure of the lumen of the distal common bile duct was confirmed under direct vision.

A definitive diagnosis of cystic BA was made, and hepaticojejunostomy with Roux-en-Y was therefore performed. The postoperative course was uneventful, and the baby was discharged once his jaundice disappeared 2 months post-surgery.

**Fig. 3:**
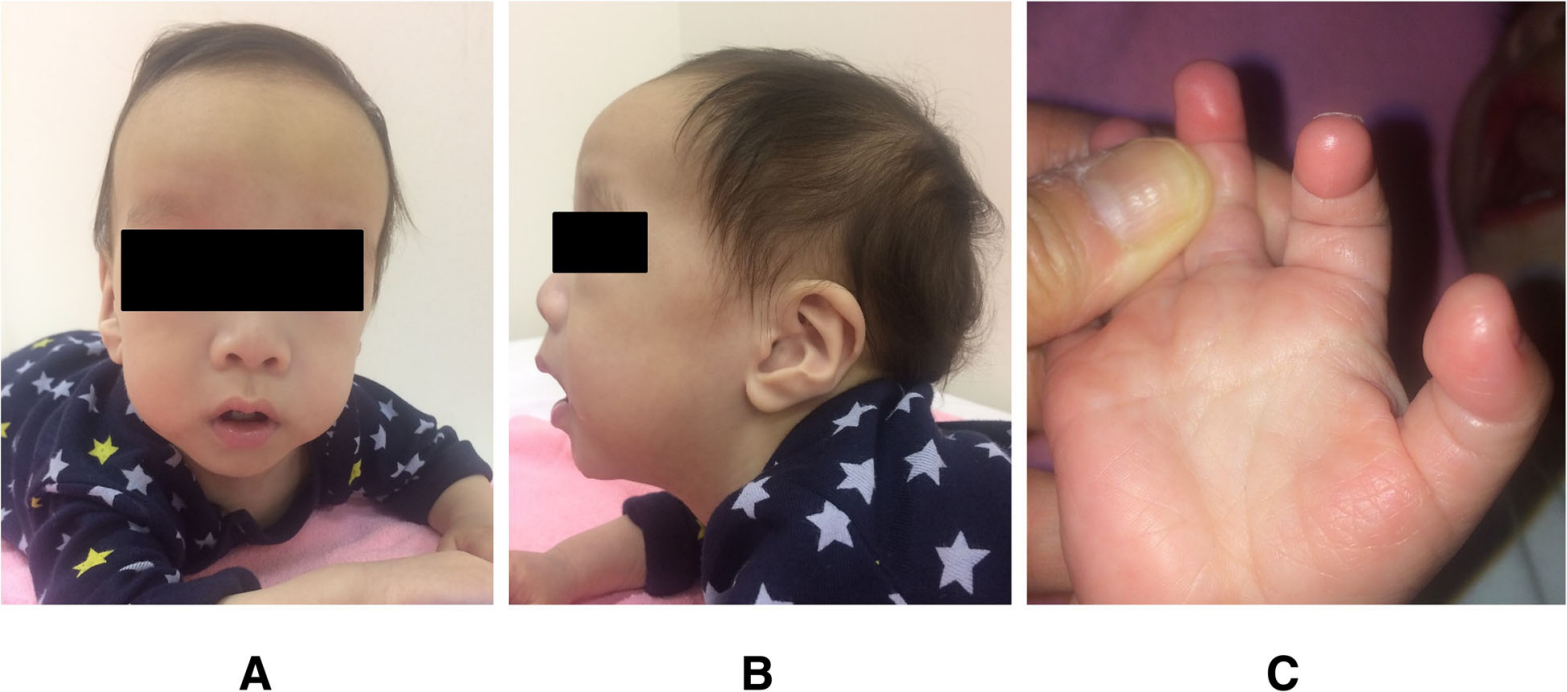
**a**–**c** Facial photographs and fetal pads photograph. Our patient had unusual facial features, including a broad forehead, orbital hypertelorism, a small, pointed chin, and large anteverted ears. He also had fetal fingertip pads on all fingers

**Fig. 4:**
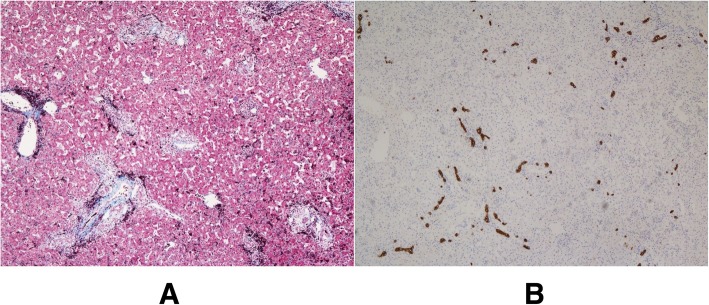
**a** Histological findings of the liver biopsy. Liver biopsy stained with Masson’s trichrome showed a portal area with mild inflammation and fibrosis. (Masson’s trichrome, original magnification × 100). **b** Immunohistological findings of the liver biopsy. Immunohistological findings of the liver biopsy for cytokeratin 7 showed interlobular bile duct paucity adjacent to the hepatocytes. (cytokeratin 7, original magnification × 100)

**Fig. 5:**
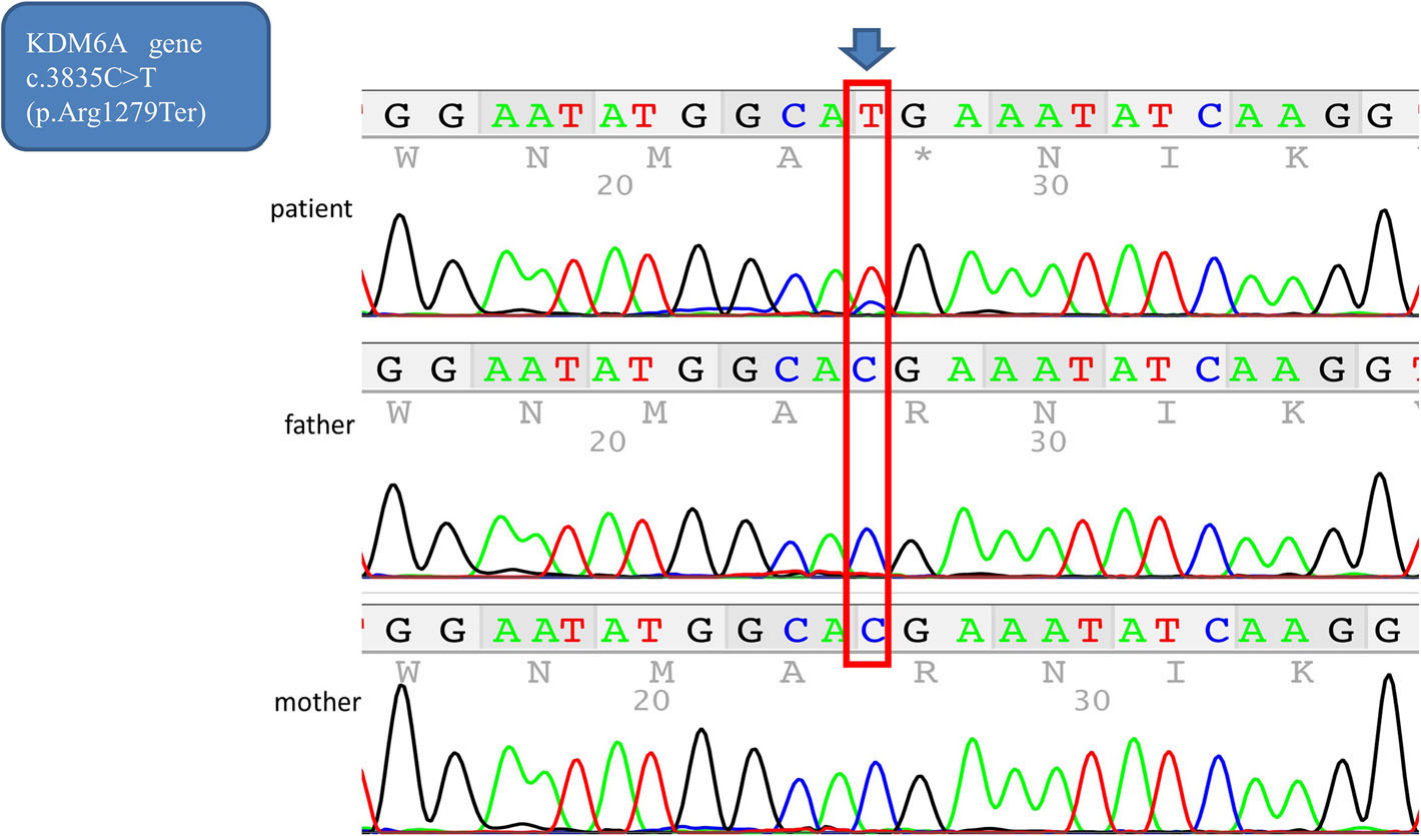
Sequencing analysis of *KDM6A. KDM6A* sequencing identified a de novo pathogenic heterozygous nonsense mutation in exon 29; c. 3835C>T (p. Arg1279Ter)

Apart from the clinical finding of BA, the baby had unusual facial features, including a broad forehead, orbital hypertelorism, a small, pointed chin, and large anteverted ears. Other findings were abnormal extremities with fetal pads (Fig. 3a–c). The skeletal radiograph was normal, and neurological examination revealed opisthotonus. Cerebral magnetic resonance imaging showed no abnormalities, and cardiac ultrasonography detected pulmonary artery stenosis and bicuspid aortic valve. An eye examination demonstrated posterior embryotoxon. Although the operative finding suggested BA, the baby had clinical features consistent with Alagille syndrome (ALGS). A liver biopsy showed a portal area with mild inflammation and fibrosis, as well as a paucity of interlobular bile ducts adjacent to the hepatocytes (Fig. 4a, b). However, *JAG1* and *NOTCH2* gene mutations could not be detected. Therefore, we performed whole-exome sequencing on DNA from his parents to examine the possibility of other syndromic disorders, and identified a de novo, novel variant in *KDM6A*. *KDM6A* sequencing identified a de novo pathogenic heterozygous nonsense mutation in exon 29; c. 3835C>T (p. Arg1279Ter) (Fig. 5). Although the clinical findings showed typical Kabuki-like phenotypes, without the face appearance, experienced clinical geneticists in our hospital could not confirm this diagnosis. When the patient was 2 years old, his biological and clinical hepatic parameters were found to be normal.

## Discussion

BA is found in nearly one-third of all neonates and infants with cholestasis and is characterized by destructive inflammatory obliterative cholangiopathy that results in varying lengths of both the intrahepatic and extrahepatic bile ducts [11]. The histopathological features of BA overlap with those of other pediatric liver diseases and rarely overlap with those of other genetic disorders and syndromes; therefore, accurate diagnosis of BA is challenging. It is crucial to differentiate between cystic BA and choledochal cyst when a portal cyst is found in a fetus [12]. In general, the size of the cystic lesion in cystic BA remains constant throughout the fetal period, while that in choledochal cyst increase with the gestation [13]. However, it is not easy to distinguish between these two diseases in neonatal or early infantile period in spite of the several diagnostic tests. Therefore, most of the child with a portal cyst detected by the fetal ultrasonography requires the exploratory laparotomy to establish a definitive diagnosis [14]. However, its timing is still controversial [15]. We could not differentiate between cystic BA and choledochal cyst. Cyst size appeared to remain unchanged during the prenatal period and at birth, whereas persistent jaundice, an acholic stool, and the elevation of liver function test value including total bilirubin, direct bilirubin, AST, ALT, γ-GTP, and TBA after feeding were observed in our case. Therefore, we decided to conduct exploratory laparotomy in early neonatal period.

ALGS is a complex, multisystem autosomal dominant disorder with incomplete penetrance that is difficult to distinguish from BA during the neonatal period. ALGS is defined clinically by the association of at least three of the five major features: chronic cholestasis, congenital heart disease, skeletal anomalies (typically butterfly vertebra), ocular abnormalities (primarily posterior embryotoxon), and distinct facial features, and genetically by heterozygous mutations in *JAG1*, or rarely, in *NOTCH2* [16–18]. Posterior embryotoxon, which was originally thought to be the characteristic ophthalmologic feature of ALGS, is found in approximately 22% of the normal population; therefore, it is now thought to have a low predictive finding for ALGS [19]. The most common initial presenting sign of ALGS in infancy is conjugated hyperbilirubinemia, which can be difficult to distinguish from other causes of obstructive cholestasis, especially BA. Regarding hepatic histopathological findings, a paucity of interlobular bile ducts is a common finding in ALGS. However, ductular proliferation is present in a small number of infants with ALGS, leading to significant diagnostic confusion. Because of the variability in the early histopathology of the liver in ALGS, a number of patients have been misdiagnosed with BA [18]. Conversely, Yamaguti et al. and Raweily et al. reported evidence of some loss of intrahepatic ducts in 20% and 16.2% of patients with extrahepatic BA, respectively [20, 21].

The present case showed three out of the five major features of ALGS, with no vertebral or characteristic facial findings. He had cholestatic jaundice, unusual facial features, posterior embryotoxon, and bile duct paucity as demonstrated by histological study. Therefore, ALGS was strongly suspected, whereas *JAG1* and *NOTCH2* mutations were not identified. Pathological findings in the present case might suggest the possibility of a transition from interlobular bile duct paucity to BA.

A gene mutation in *KDM6A* has been identified in KS, a rare disorder also known as Kabuki makeup syndrome or Niikawa–Kuroki syndrome. KS is generally diagnosed clinically based on the combination of five main criteria: (i) postnatal growth retardation; (ii) development of mental disability; (iii) typical facial features; (iv) skeletal anomalies; and (v) fetal fingertip pads [5]. Congenital heart defects are observed in approximately 40–50% of KS patients according to the literature [22]. Two causative genes have been identified in KS patients. In particular, a mutation in *KMT2D* at 12q13.12 accounts for 55–80% of KS cases, whereas 9–14% of *KMT2D*-negative patients have a deletion or mutation in *KDM6A* at Xp11.3 [23]. Bögershausen et al. reported on a KS patient with a *KMT2D* mutation who presented with neonatal cholestasis with bile duct paucity in addition to the typical clinical features of KS, although their case did not show pathogenic variants in either *JAG1* or *NOTCH2*; they hypothesized that the *KMT2D* mutation might have affected several key Notch signaling components [24]. *KDM6A* encodes lysine-specific demethylase 6A (UTX), a JmjC domain-containing protein that specifically demethylates the H3K27me3/me2 mark [25]. Further, Jin et al. reported that genes encoding some upstream activators of NOTCH are highly enriched for H3K27me3 [26]. However, to date, there is no known connection between *KDM6A* and the Notch signaling pathway. Zagory et al. demonstrated increased activation of the Notch signaling pathway in BA patients [27], and hepatic malformations have so far been described in three KS patients with extrahepatic BA despite no gene examinations [7, 9, 10]. Notch signaling pathway has a crucial role in vascular development and specific roles in the differentiation of biliary epithelial cells [28]. It is thus conceivable that the orbital vascular and bile duct malformations might be due to the embryological signaling defect.

The present case had distinctive clinical findings such as facial anomalies, fetal fingertip pads, and mental retardation, although the skeletal and facial anomalies were not typical; he also had a gene mutation in *KDM6A* and bile duct paucity. Recently, KS tends to be diagnosed by the KDM6A mutation via whole-exome sequencing rather than by the clinical finding of KS. However, KDM6A mutation does not always lead to the definite diagnosis of KS in the case not having the distinctive clinical feature [29]. Although our patient had KDM6A mutation and some clinical features of KS, it was clinically difficult to diagnose without characteristic facial features. The experienced clinical geneticists in our hospital could not confirm this diagnosis.

Based on the interesting findings of these previous reports and the present case, liver anomalies may be regulated by *KDM6A* mutations.

## Conclusions

We encountered a rare neonatal case of cystic BA with the histological finding of interlobular bile duct paucity and a genetic mutation in *KDM6A*. Additionally, several clinical findings consistent with both ALGS and KS made it difficult to confirm the diagnosis of BA. In neonates with syndromic neonatal cholestasis or liver disease of unknown cause, exome sequencing could be useful to confirm the diagnosis. Based on the interesting findings of the present case, we hypothesized that *KDM6A* might be associated with hepatic malformations via a connection with the Notch signaling pathway. Further genomic investigations are warranted in BA patients with overlapping clinical and histological findings with ALGS or KS to elucidate the mechanism of hepatic malformation, such as a possible connection between *KDM6A* and the Notch signaling pathway.

## Data Availability

Data sharing is not applicable to this article.

## Abbreviations

ALGS: Alagille syndrome
BA: Biliary atresia
KS: Kabuki syndrome
